# Learned uncertainty: The free energy principle in anxiety

**DOI:** 10.3389/fpsyg.2022.943785

**Published:** 2022-09-06

**Authors:** H. T. McGovern, Alexander De Foe, Hannah Biddell, Pantelis Leptourgos, Philip Corlett, Kavindu Bandara, Brendan T. Hutchinson

**Affiliations:** ^1^School of Psychology, The University of Queensland, Brisbane, QLD, Australia; ^2^School of Educational Psychology and Counselling, Monash University, Melbourne, VIC, Australia; ^3^Department of Psychiatry, Yale University School of Medicine, New Haven, CT, United States; ^4^School of Psychological Sciences, The University of Melbourne, Melbourne, VIC, Australia; ^5^Research School of Psychology, The Australian National University, Canberra, ACT, Australia

**Keywords:** anxiety, free energy, active inference, belief, predictive coding, perception, clinical, psychopathology

## Abstract

Generalized anxiety disorder is among the world’s most prevalent psychiatric disorders and often manifests as persistent and difficult to control apprehension. Despite its prevalence, there is no integrative, formal model of how anxiety and anxiety disorders arise. Here, we offer a perspective derived from the free energy principle; one that shares similarities with established constructs such as *learned helplessness*. Our account is simple: anxiety can be formalized as *learned uncertainty*. A biological system, having had persistent uncertainty in its past, will expect uncertainty in its future, irrespective of whether uncertainty truly persists. Despite our account’s intuitive simplicity—which can be illustrated with the mere flip of a coin—it is grounded within the free energy principle and hence situates the formation of anxiety within a broader explanatory framework of biological self-organization and self-evidencing. We conclude that, through conceptualizing anxiety within a framework of working generative models, our perspective might afford novel approaches in the clinical treatment of anxiety and its key symptoms.

## Learned uncertainty

Nature consists of dynamic and complex systems (Friston, 2010; Zednik, 2011). For a biological system to exist, it must have the capacity to maintain its own boundaries—lest it cannot be distinguished from other systems. A fundamental property of any biological system is therefore the requirement that it can individuate itself from its environment. The free energy principle asserts that to do this, biological systems model external states and themselves within those states (Friston et al., 2006; Friston, 2010). This occurs through a process where the system samples information from outside its boundaries, *via* its suite of sensory channels, and acts based on that sampled information, under some world or generative model. Ultimately, this is an iterative process in which the nature of what is perceived of the external state (*sensory impressions from the world*) informs the system’s internal state. Internally, the system then generates a model of the external state of affairs, which informs how the system ought to act on the world. Via action, the system influences its external state, which leads to sensory feedback about the system’s world model—and the “perception-action” cycle repeats.

## The free energy principle: A primer

The free energy principle describes how biological systems resist dissipation and destruction (Friston et al., 2006; Friston, 2010). Under thermodynamic principles, all systems move toward disorder (Schneider and Kay, 1994; Hirsh et al., 2012). Yet, biological systems resist dissipation and instead maintain themselves in viable states that underwrite their survival (Hirsh et al., 2012; Ramstead et al., 2018). In fact, biological systems restrict themselves to a relatively small set of such “attractor” states, the spectrum of which can be thought of as equivalent to homeostasis: a variable number of states within which the system can feasibly sustain its own existence. The crucial insight afforded by the free energy principle is that this process—of continuously moving toward attractor states—is an intrinsic property of biological systems that can be described as emerging *via* modeling the “sensed” world (Friston, 2010; Hirsh et al., 2012). Broadly then, the free energy principle is about how biological systems self-fulfillingly define themselves as systems *per se*, and in doing so move away from destruction and toward attractor states.

### What is free energy?

To “minimize free energy” is to minimize error (or surprise) engendered by exchange with the external milieu (including one’s own body). Free energy can be considered a proxy for surprise, where this kind of surprise (a.k.a., self-information) can be read as the (negative logarithm of the) evidence for the system’s model. The system continually minimizes surprise, and in so doing attempts to minimize uncertainty (i.e., expected surprise) in its sensory exchanges with the world. Yet, no model will perfectly capture the external world it is modeling and will therefore have to deal with uncertainty. To be sure, some degree of uncertainty is a requisite for system optimization; otherwise, our internal representation would no longer be amenable to the accommodation of *change* in a capricious and itinerant world. However, too much uncertainty or error inherently contradicts the system’s goal of restricting itself to its attractor states—those that characterize the kind of thing that it is (Bruineberg and Rietveld, 2014; Badcock et al., 2017, 2019; Bruineberg et al., 2018; Ramstead et al., 2018). The free energy principle thus specifies that free energy must be actively upper bounded to ensure expected surprise (uncertainty) is minimized or model evidence (marginal likelihood) is maximized (Friston, 2010; Ramstead et al., 2018). Free energy is the system’s most accurate estimation as to the uncertainty that exists “out there,” and minimizing free energy is akin to minimizing the error in the system’s prediction about the world. To “minimize free energy” is thus to maximize precision in the system’s capacity to model its own world. This can be neatly summarized as self-evidencing (Hohwy, 2016).

Models of perception rooted in prediction are generally considered to have stemmed from Helmholtz (1860) notion of unconscious inference. Helmholtz (1860) proposed that we attempt to infer our environment *via* unconscious cues, and constructivist theorists including those within Jean Piaget’s (developmental schema theory, see Piaget, 2003; Feldman, 2004; Beard, 2013) and George Kelly’s (personal construct psychology, see Kelly, 1955) schools of thought later posited similar notions from the top-down cognitivist perspectives. Variations in earlier schools of thought can be seen throughout literature, with some models having emphasized the conceptual representation of cognition; others focusing on the developmental impact of learning (e.g., models following from Jean Piaget and Lev Vygotsky); and yet others focusing on the state and process changes that occur in one’s mental representation (e.g., MacKay, 1956; Neisser, 1967; Gregory, 1980; Yuille and Kersten, 2006). We focus on the latter of these here, with an emphasis on top-down inferences about one’s environment.

State-based predictive models have evolved over time (McClelland and Rumelhart, 1981; Rao and Ballard, 1999) and are generally considered under the scope of “predictive processing.” Predictive processing equates the brain to that of a scientist—it makes observations, collects data from the external environment and generates and updates workable hypotheses based on the current data available (Hohwy, 2013, 2017). From a neuroscientific standpoint, predictive processing recasts the classic idea of the brain as an information processor, specifying instead that top-down and bottom-up neural networks are functionally driven toward signaling prediction and the consequent minimization of prediction error (Friston, 2010; Clark, 2013). Top-down neural activity associated with reentrant loops—those that provide feedback from higher level brain regions to sensory processing areas—are conceptualized as propagating a prediction about a given sensory input, given some high-level representation or prior expectation about its cause. The bottom-up or feed-forward activity associated with sensory input then carries the ensuing prediction error. If the (top-down) prediction sufficiently accounts for the (bottom-up) signal, the error is “explained away”—for example, the signal is attenuated *via* inhibitory mechanisms (Friston, 2012b; Hohwy, 2013, 2017). If the prediction *cannot* sufficiently account for the (bottom-up) signal, the error propagates up the hierarchy and the (top-down) predictive model is revised (Friston, 2008; Friston and Kiebel, 2009; Huang and Rao, 2011; Bastos et al., 2012; Alexander and Brown, 2018). Formally, this can be described in terms of Bayesian belief updating, where neuronal message passing involves the reciprocal exchange of top-down predictions and bottom-up prediction errors (Friston et al., 2017). In this way, signals cascading bidirectionally throughout the cortical hierarchy can be thought of as building (hierarchical) generative models about the sensed world.

### Predictive processing and the free energy principle

The free energy principle and predictive accounts describe and seek to explain the processes by which the brain functions. Predictive processing can be read as an application of the free energy principle—one that commits to a specific implementation, usually in terms of the predictive coding schemes described earlier. These applications describe the brain as a system that continually models the world in order to understand it. The free energy principle provides a description of the fundamental laws underlying biological systems, whereas predictive processing is more typically leveraged as a process theory for psychology and situated cognition (Friston and Kiebel, 2009; Bogacz, 2017). Simply put, the free energy principle is concerned with systems, whereas predictive processing applies the free energy principle—as a method—to specific systemic and functional hierarchies in the brain.

Though primarily concerned with system dynamics, the free energy principle can be applied to biological phenomena at every scale—from the microscopic to the psychological. At the level of human psychology, belief formation offers a sound illustration: under the free energy principle, “beliefs” correspond to probability distributions over external states parameterized by internal representations of those states, which the individual develops based on observing and modeling the world (and themselves within the world, a point we return to later in the section “Learning Uncertainty”). Belief formation is a useful example, as it allows us to illustrate the synonymity between the free energy principle and predictive processing—predictive processing describes belief formation through the updating and development of *priors*, in that beliefs are formed *via* the brain learning about the world based upon *prior* observations of the world; a classic example being a belief that water quenches thirst is formed based on prior observations that water quenched thirst—or put more precisely, based upon the prediction errors of a generative model that did not predict the thirst quenching consequences of water. As a technical note, while debate centers on how such predictions are optimized (see Bowers and Davis, 2012), generally Bayes’ theorem is considered a formal expression of how predictions are optimized in terms of Bayesian belief updating. This process of belief updating “just is” the process of self-evidencing detailed earlier (for the mathematically minded, refer to Friston and Kiebel, 2009; Spratling, 2016; Bogacz, 2017; Sterzer et al., 2018).

The free energy principle and predictive processing are therefore related in how they describe belief formation in terms of learning and perceptual inference—the difference is that the free energy principle provides a principle or “method” that underlies and attempts to dissolve disciplinary boundaries (a strength that should not be understated, Friston (2012a, 2019); Rubin et al., 2020). In summary, these approaches together not only explain optimal prediction and model generation but allow us to consider how contextual cues may lead to differing probabilities of a given state or outcome. Crucially, we now consider the outcomes consequent on decisions, choices and action.

### Active inference

Cognitive systems are not passive observers of the world. Instead, cognitive agents act and sample the environment to test their predictions about the causes of sensory data. Derived from the free energy principle, active inference describes how agents seek to minimize variational free energy through testing and updating generative models *via* sequences of actions predicted to result in preferred outcomes (i.e., *action policies*, see Smith et al., 2022). Active inference assumes that agents carry preferences for what states they prefer to occupy; namely, those that minimize uncertainty or expected surprise, where a surprising state of being is—by definition—aversive (Smith et al., 2022). Action policies and subsequent updating of generative models are thus driven toward the attainment of preferred sensory outcomes, and the avoidance of non-preferred outcomes.

In active inference, agentic preferences over sensory outcomes are typically leveraged as prior predictions, called prior preferences. Insofar as the sensory outcome ultimately diverges from preferred outcomes, this will be surprising (Smith et al., 2022). As agents make decisions about possible action sequences, the agents calibrate the amount of expected surprise that should be generated by one course of action vs. another. Once this calibration is complete, agents can then infer what they are most likely to do. This is sometimes described as planning or control as inference (Attias, 2003; Botvinick and Toussaint, 2012; Millidge, 2019). In this way, preferred outcomes are obtained with action policies *via* minimizing the expected divergence between preferred sensory outcomes and those anticipated when committing to a particular plan. The key point here is that actions will be chosen based on the agent’s estimation of how likely they are to generate preferred sensory outcomes, which oftentimes is those which are consistent with the agent’s current world model (Smith et al., 2022).

## Computational approaches in psychiatry

In recent years, there has been an increase in the use of computational methods in psychiatry research, both as methodological tools and for a mechanistic and conceptual understanding. We focus on the latter. Theoretical instantiations of the free energy principle such as predictive processing and active inference are increasingly utilized for understanding a range of psychiatric disorders, including Post Traumatic Stress Disorder (Linson and Friston, 2019), stress (Peters et al., 2017), eating disorders (Barca and Pezzulo, 2020), obsessive compulsive disorder (Fradkin et al., 2020), anxiety (Clark et al., 2018), and depression (Barrett et al., 2016).

Recent work has focused on belief updating in anxiety disorders, such as social anxiety (Smith et al., 2019a; Gerrans and Murray, 2020), and negative mood and affect (Joffily and Coricelli, 2013; Van de Cruys, 2017; Hesp et al., 2021). Broadly, these works interpret anxiety disorders as encoded uncertainty: agents encode and act upon beliefs that the world is unpredictable and uncertain, meaning that action policies cannot sufficiently minimize expected surprise or uncertainty (see Mathys et al., 2014; Clark et al., 2018; Smith et al., 2019a, 2021b; Fradkin et al., 2020). For example, Clark et al. (2018) describes anxious depression as expected unpredictability (predictions with low precision)—that is, an agent will act to minimize expected surprise, but cannot reliably do so. The sense of expected unpredictability precludes the effective resolution of uncertainty, given an agent cannot infer how she should act.

It is important to note that uncertainty is not necessarily linked to negative affective valence. Expected uncertainty can elicit positive emotions such as the excitement associated with novelty, i.e., the opportunity to learn and reduce uncertainty (Anderson et al., 2019). Instead, as described by Hesp et al. (2021), “agents infer their valence state based on the expected precision of their action model” (p. 398). Thus, negatively valenced uncertainty characteristic of anxiety occurs when the agent infers that their current action policies are *not* sufficient to resolve uncertainty—a point we expand on later (for a fuller discussion, see Anderson et al., 2019). In what follows, we discuss traditional cognitive accounts of anxiety, before turning to how the free energy principle and active inference might furnish further insight on anxiety formation.

### Anxiety

Traditional models of (generalized) anxiety are underpinned by the notion that erroneous beliefs lead to perpetuated and often exaggerated anxiety responses to a situation or context. Early behavioral models of anxiety relate to conditioning or learning (Clark, 1986, 1999; Beck and Clark, 1988). Entering a situation in which panic or anxiety has been learned leads to subsequent anticipation of anxiety upon re-entering that situation, which provokes further anxiety (Clark, 1986). The work of Beck and Ellis established common cognitive biases and filters that are thought to skew one’s perceptions and conceptions of various circumstances (Beck, 1970; Ellis, 1980). Common examples include catastrophizing, dualistic thinking, and exaggeration of anxiety-provoking stimuli (Beck and Weishaar, 1989; Benjamin et al., 2011). The later stimulus-response model of anxiety is compatible with the traditional cognitive framework, as entrained behaviors may exacerbate cognitions and vice versa. For example, Clark’s (1986) cognitive model proposes panic attacks are a “catastrophic misinterpretation” (p. 462) of bodily sensations that amounts to a cyclical feedback response in instances when anxiety is expected.

While the cognitive-behavioral model has informed clinical practice for decades (for an excellent overview, refer to Behar et al., 2009), it is not without its critics. One of its major criticisms in relation to the treatment of anxiety disorders relates to the observation that panic sensations and stress-related responses can persist even though erroneous beliefs have been adequately challenged (Beidel and Turner, 1986; Cartwright-Hatton et al., 2004; Linden et al., 2005). For example, a therapist may convincingly establish (with a client) that the client’s thoughts about being made fun of during public speaking are highly exaggerated and unlikely to be true, yet the client persists with a sense of dread and anxiety when confronted with the task of public speaking (Gerrans and Murray, 2020).

We should first point out that numerous theorists have proposed unique means of conceptualizing beliefs centric to anxiety that challenge traditional models such as CBT and rational-emotive behavioral therapy. For instance, rather than interpreting beliefs as static filters that inform one’s perceptions in a similar manner in all situations, Kelly (1955) proposed that erroneous beliefs have a tangible “weight” and might be conceived of as tight/loose or brief/elaborate, among other construct-based corollaries. Conversely, emotion-focused therapies (EFT) argue that affect precedes belief formation, and hence is ultimately subject to factors which determine emotion (Greenberg, 2004). Here, we argue that a view positioned within the free energy principle and active inference may afford opportunities to re-conceptualize anxiety formation within the framework of working generative models, rather than static beliefs or filters. Our primary divergence from extant work is that we argue for the process of how an agent who is initially without anxiety (i.e., believes her actions will lead to preferred outcomes that minimize uncertainty) may learn and update *beliefs about uncertainty*, and thus form symptomology consistent with generalized anxiety. The approach we propose therefore neither favors earlier biological and behaviorist theories of anxiety disorders, nor later cognitivist accounts, but aims to unify both approaches by explaining bottom-up belief propagation within a systemic optimization framework.

### A novel perspective *via* the free energy principle?

The free energy principle provides a useful framework to understand the *what* and *how* of anxiety formation. Under the free energy principle, anxiety can be described as discerned uncertainty about whether actions will minimize uncertainty, forged *via* sufficient exposure to surprising outcomes (Hirsh et al., 2012). That is, within a biological system that strives toward attracting states, anxiety is the psychological consequence of an irreducible *mismatch* between the predicted consequence of actions and the outcomes encountered, meaning uncertainty about action policies is irreducible. Sufficiently long-lasting and persistent uncertainty of this sort impairs the agent’s capacity to develop *adaptive* models (i.e., models that afford effective sampling and actions for the minimization of expected free energy). When this occurs, the perception-action cycle becomes (dysfunctionally) geared toward unpredictable outcomes, which affirm and reinforce a world model in which uncertainty is the norm. The system thus learns to expect uncertainty in future iterations of the perception-action cycle. This, we suggest, is *learned uncertainty*, a process that is especially pernicious because it precludes its own “adaptive” resolve. In other words, “*if everything I do leads to uncertain outcomes, then this is a good model for my lived world—and there is no reason to change this model*” (c.f., learned helplessness).

Based on the free energy principle, the sustainable existence of a biological system is tantamount to it returning to a small number of attracting states. Despite the system developing an uncertain model, then, its goal and action policies are still driven toward the minimization of free energy—otherwise, as a system, it dissolves into states that are no longer characteristic of the system in question (e.g., death, dissipation, decay, etc.). The biological system is thus left in a position where it is driven toward free energy minimization, based upon the prior belief that its behavior will resolve the uncertainty and enable the agent to secure preferred sensory outcomes. Anxiety formation can thus be considered learned uncertainty *about* the sufficiency of the agent’s world model and action policies in bringing about preferred sensory outcomes. This uncertainty, we argue, is learned from feedback about the efficacy of action policies for effectively reducing uncertainty. Despite experiencing this uncertainty, the system must still attempt to minimize it, but it does not have an adaptive way of doing so, given its conviction that the world is unpredictable and there are no policies at hand to resolve this uncertainty. Technically, this reflects the fact that expected free energy entails an epistemic aspect; namely, the resolution of uncertainty through expected information gain of the kind that underwrites novelty. However, the epistemic affordance of novelty disappears in a capricious and unpredictable world.

Overall, then, the existence of persistent anxious states—as a characteristic of anxiety disorder—is analogous to a system learning uncertainty through model updating and action policies. Put differently, given enough sampling of an uncertain world, the system learns that this kind of uncertainty is irreducible (also see Mathys et al., 2014). This is consistent with experiential accounts of anxiety, in which individuals with anxiety disorder often report the world as an unsafe and unpredictable place; whereas those with lower anxiety levels are more likely to report their account of the external world as a safe and trustworthy place (Wells, 1999). A biological system, having had persistent uncertainty in its past, will be more likely to expect ambiguous feedback as to how effective its world model and action policies are in reducing uncertainty in the future. This occurs irrespective of whether that uncertainty is reducible, meaning the expected uncertainty will be disproportionate to the actual uncertainty that could be resolved (given an alternative repertoire of policies). In other words, the system learns its model (and action policies) are insufficient for minimizing uncertainty, resulting in the psychological experience consistent with generalized anxiety. From the perspective of affective or emotional inference, the psychological experience in question can be thought of as another part of the generative model that best explains the state of affairs: “*I must be anxious because I cannot decide what to do.*” In other words, experienced anxiety reflects the fact that particular biological systems have sufficiently expressive generative models to recognize that they are in a state of uncertainty.

## Learned uncertainty at the flip of a coin

An example from probability theory helps illustrate learned uncertainty. Let us suppose an agent carries what they think is a weighted coin, such that 9 out of 10 times it will result in heads. Every time it lands on heads, the agent receives a reward. In this example, the agent expects that flipping the coin 10 times will result in sampling approximately 9 observations of heads. As such, the agent carries a model of preferred observations (i.e., the coin landing on heads, given it leads to a reward), and an idea of what action policy will enable it to return to that state (i.e., flipping the coin).

From here, we can imagine (very broadly) three informative scenarios for the agents’ preferences and action policies. First, the coin lands on heads 9 out of 10 times. In this scenario, the agent obtains its preferred sensory observations, and thus learns that merely flipping the coin is sufficient for obtaining these preferred observations. Second, in a drastic departure from what the agent expects, the coin lands on tails 9 out of 10 times, violating both its prior preferences and precluding preferred observations. In this scenario, the sensory feedback, being highly antithetical to the agents preferred states, inform the agent that they must update their world model and action policies in order to obtain the reward in future instances (and hence minimizing uncertainty over their world model and action policies). In predictive processing terms, we might say that the prediction error (elicited by the coin landing on tails) propagates upward through the cortical hierarchy, allowing the agent to update their expectations and action policies.

In a third scenario, we can imagine that the coin lands on heads a random number of times. This scenario offers an illustrative case of what we refer to as *learning uncertainty*. In this scenario, the agent cannot be either sure or unsure of the efficacy of its world model. Remember that the agent started with the expectation that their action policy (flipping the coin) would bring about preferred sensory outcomes (the coin lands on heads 9 time). In this example, the agent expected that flipping the coin would result in attainment of preferred observations because of their action policies. However, here, this “prior over policies” is partially supported, but also partially refuted (depending upon the outcome). This would therefore generate some prediction error (given that the information sampled suggests the agent was in error in expecting 9 heads), but the key point is that the divergence between the observed outcome and predicted outcome is not sufficient to outright refute the system’s model (and action policies), nor prompt its complete reevaluation.

If the agent were to flip the coin 10 more times, they cannot be sure whether their current world model (*the coin is weighted toward heads*) or their action policies (*flipping the coin*) are sufficient for reliably bringing about preferred sensory outcomes (the coin lands on heads more often than tails). This means that the agent is left with uncertainty regarding (a) whether their model of the external world is sufficiently accurate and (b) whether the actions they undertake are sufficient for obtaining preferred sensory outcomes. If we were to, say, flip the coin 100 times, the feedback becomes clearer: “*my model is right sometimes, but wrong sometimes. No matter what I do, I cannot be sure how the coin will land in future.*” In animal studies, rodents shocked 50% of the time (Zhang et al., 2019; or at random, Seidenbecher et al., 2016) show higher levels of anxiety compared with mice shocked at more predictable rates (e.g., 0 or 100% of the time). These findings are precisely what we are referring to—agents given inconsistent sensory data regarding the efficacy of their world model and action policies will be more likely to generate and act upon anxiety.

One might be quick to point out that, in our example scenario, the agent might be wise to update their “belief” that the coin is weighted—after all, the flip of a coin is best expressed as a discrete uniform distribution: only two possible outcomes exist, and the probability of heads or tails is theoretically equal. Hence, a situation in which the coin lands on heads approximately 50 times out of 100 would fit with the appropriate behavior of a standard unweighted coin. We address this more explicitly below (subsection “Learning Uncertainty”). For present purposes, keep in mind this is merely an example—our point here is *not* that sometimes modeling uncertainty is accurate, but rather that the observation has diverged sufficiently to generate uncertainty in the system’s own model, but not sufficiently to lead to model updating. More generally, what we are talking about is the kind of radical uncertainty or ambiguity that precludes “useable” or informative data being sampled from its environment. In free energy terms, the system’s predictions are guided by an uncertain probability distribution in which all possibilities are approximately equal (or otherwise cannot be differentiated), and thus the system’s actions are guided by a model that performs at chance. We consider this the initial “lever” for anxiety formation.

### Learning uncertainty: Where hierarchical models go wrong

To fully appreciate this process requires appeal to a higher-order level in which the system makes predictions based on hierarchical world models. In active inference, hierarchical models describe the correspondence between different levels of representations (i.e., Bayesian beliefs) that an agent may hold about the world. These are often referred to as *deep temporal models* (Friston et al., 2018; Smith et al., 2019a,b). Such schemes demarcate between “level 1” and “level 2” models of the world, where level 2 models store posteriors inferred by level 1 models. What this means is that level 2 models evolve over slower timescales than level 1 and are hence more impervious to current sensory data. An intuitive example is provided by Friston et al. (2018), where an agent reads a passage of text. In this example, a level 1 model makes an inference about individual words being read, while level 2 models infer the overall direction of the text passage. This type of hierarchical model explains how agents infer state transitions over nested time sequences (Smith et al., 2022), in that they consider accumulated experiences in inferring a current context, and how current contextual states correspond to higher levels of the model.

Returning to our example, suppose we now have one hundred observations cataloged into 10 sets of 10. Now imagine the agent continues to observe in each sequence that the coin lands on heads an unpredictable number of times. In this case, the agent is offered consistent sensory feedback that their model of the world is not sufficient for reliably inducing preferred observations, but not wholly insufficient, either (keeping in mind that they are still expecting 9 out of 10 heads). Over the entire sequence of flips (i.e., the 10 set of 10 flips), the second level of the model “learns” that action policies are not able to reliably bring about preferred sensory outcomes but are also not insufficient to the extent that the agent must not exhibit wholesale change to their action policies. If the agent consistently observes feedback that their model is not sufficient for reliably inducing preferred sensory outcomes, they will be in a position where the second level of their model is not all that informative for inferring the hidden causes of current sensory states (i.e., observations at level 1). Instead, they are left in a position where they are neither sure nor unsure on the utility of their action policies. This, we argue, is a starting point for the proliferation of anxiety disorder. We can understand this *via* reference to Bayes rule: priors update over time in accordance with new information, from which predictions are made about what will be subsequently observed (refer to Westbury, 2010; Friston, 2012b; Mathys et al., 2014). Anxiety disorder in this example therefore forms *via* Bayesian learning: the system optimally predicts that there is uncertainty, and that their action policies cannot reliably induce preferred sensory states.

To be precise, momentary anxiety can be thought of as generated *via* high entropy probability distributions within a single perception-action cycle, whereas anxious “beliefs” form based upon low entropy probability distributions of those “observations of observations” (i.e., *meta* observations, or level 2 of the hierarchical model), the composition of which are more uncertain probability distributions. This account shares similarities with several previous frameworks. For example, Chekroud (2015) suggested the formation of depression can be Bayes *optimal* (i.e., learned helplessness), where depressive beliefs form despite a match between information sampled and the system’s empirical priors (Chekroud, 2015; Holmes and Nolte, 2019). An agent with learned helplessness has learnt that, irrespective of their particular action policies, they cannot resolve their uncertainty—no matter what they do, they will consistently find themselves in states that depart from their preferred sensory outcomes (Seligman, 1972). Learned uncertainty departs from learned helplessness in the sense that, in the former, the (bayes optimal) agent does not have sufficient evidence (i.e., useful sensory data) to conclude helplessness but rather has modeled inconsistency in their successes in obtaining preferred sensory outcomes. The key point is that, in the case of learned helplessness, there exists certainty *inherent* in an agent reliably finding themself in states *opposed* to those they prefer.

More recently, consider Linson and Friston’s (2019) account of the formation of PTSD. They posit that PTSD is characterized by a reduced confidence in one’s ability to resolve prediction errors, given the failure to resolve these errors when experiencing a traumatic event (Linson and Friston, 2019). In the case of anxiety, we suggest that the traumatic event(s) is not necessary for its formation, merely a sufficient presence of uncertainty (or prediction error, under Linson and Friston’s model). Via this process, the organism learns an “inability” to resolve prediction errors. In this way, anxiety formation is approximately analogous to PTSD formation, but without the accompanying high precision gleaned from experiencing a traumatic event (and hence learning its own catastrophic failure to resolve prediction errors).

We suggest generalized anxiety forms because of the organism’s inability to internally model information, given some initial state of sufficient mismatch in the perception action cycle. In essence, over time, the system models its own priors and action policies, in a Bayesian *optimal* way, as not adequately updating in a way that alters the probability of a future outcome. In so doing, anxious priors’ form—beliefs pertaining to the uncertainty in the system’s world model and action policies bear disproportionate weight on top-down processes responsible for model formation, and the agent iteratively perceives the outside world as if it were still uncertain, even when it becomes more certain. Despite this, as specified by the free energy principle, the system is driven toward minimizing free energy. The biological system therefore expects that its own modeling and action policies are neither sufficient nor insufficient—and yet still necessary—and generates the belief that the inherent uncertainty will persist across time (see Dickstein et al., 2010; Grupe and Nitschke, 2013; Fonzo and Etkin, 2016; Kannis-Dymand et al., 2020; LaFreniere and Newman, 2020). External uncertainty therefore generates internal uncertainty *about* external certainty, along with the necessary action policies that would resolve it. Because this occurs in a Bayesian optimal way, model precision increases, despite clearly not being adaptive under novel environmental conditions where certainty may now exist—and *learned uncertainty* results.

It is well-established that persistent uncertainty is linked to higher formation of anxious beliefs across both short and enduring time periods (Epstein and Roupenian, 1970; MacLeod and Cohen, 1993; Chorpita and Barlow, 1998; Gutman and Nemeroff, 2003; Grillon et al., 2004; Compton et al., 2008, 2010; Murray et al., 2009; Kendall et al., 2010; Burke et al., 2017). For example, greater adversity in childhood is predictive of more severe anxiety symptoms in adulthood (Hayward et al., 2020). Broadly, this research illustrates our point thus far—uncertainty *at an early stage of the model’s development* instigates the proliferation of uncertainty at later stages of the model’s development—the downstream effects of which are (at a minimum) inhibition of adaptive priors over policies from which to operate. In other words, initial uncertainty lays the groundwork for how generalized anxiety forms.

Keep in mind that our example nulls over much of the finer detail of how modeling might occur in biological organisms (for example in models of active inference; Smith et al., 2022). Our model also does not detail with any level of fidelity the nature of the set of variables that separate the internal state of the system from the external state, in other words how the system differentiates the self from the environment (referred to as a Markov blanket, see Clark, 2017; Ramstead et al., 2018). Hence, our model does not afford (at least currently) insights into the nature of the interoceptive aspect of anxiety (i.e., modeling and predictions about the system’s own internal state, Barrett and Simmons, 2015; Paulus et al., 2019), but this will remain an important element of the model to develop in future.

### Accurate modeling of an uncertain world vs. inaccurate modeling of a certain world

A final point—as touched on above, it is important to distinguish between *accurately* modeling an environment *with* true uncertainty (as opposed to spurious prediction errors), such as in the case of an equally weighted coin (where modeling the outcomes as a discrete uniform distribution might accurately represent the external state), to *inaccurately* modeling an environment *without* true uncertainty. In some, perhaps even many, cases, the world truly is uncertain—and hence modeling an uncertain world accurately reflects the true state of affairs. In our view, this is *not* equivalent to anxiety, nor does it necessarily imbue the individual with the psychological discomfort or recognition of anxiety. However, it *is* a necessary step in the formation of anxiety disorder. It is here in which the modeling of uncertainty persists for some threshold of time that belief structures form specifying that uncertainty will persist. These belief structures are then levied in subsequent circumstances where environmental contingencies may now impart certainty. Notably, and consistent with the free energy principle, some degree of uncertainty must exist in any given system, and a level of tolerance for that uncertainty is anticipated—and indeed inferred in the form of predicted precision. However, in the case of anxiety syndromes, the individual has learned uncertainty will persist, and hence now *inaccurately* models (a world of) uncertainty. Rather than returning to the prior model’s homeostasis, a new homeostasis, modeled on uncertainty, is thus formed (e.g., at a deeper modeling layer). The departure point for pathological anxiety occurs when the world returns to certainty, yet because uncertainty is now positioned within the agent’s model and action policies, the agent still behaves as if the world is uncertain—and never learns otherwise.

## Criticisms and limitations

Perhaps the biggest strength of our account is that it nests anxiety within a broader framework offered by the free energy principle and active inference, and hence allows for an explanation of anxiety formation operating from first principles. Crucially, this means that one can simulate the proposed belief updating processes *in silico* and, in principle, use these simulations to fit observed choice behavior in the spirit of personalized medicine (Schwartenbeck and Friston, 2016; Smith et al., 2021a,b). This remains a key endeavor we seek to address in future. Despite this strength, we must stress that our proposal is not intended to be an exhaustive account of anxiety (for example its long-term clinical presentation). Rather, it merely aims to describe how anxiety and anxiety disorder might form. Still, there are several key objections that can be anticipated, and which require addressing.

### What can learned uncertainty offer beyond other accounts of anxiety?

Possibly the most critical objection to our proposal might be what it offers beyond other models, such as behavioral models of association and conditioning. The key difference, and its strength, comes from the specification of the free energy principle that all systems are driven toward minimizing free energy. In our account, the formation of anxiety does not (cannot) change this. Therefore, the framework can be used to generate predictions about future behaviors, specifically, in relation to how anxiety will interact with the processes laid out by active inference for how systems go about minimizing free energy. In doing so, our model may even point to new treatment options. The approach proposed here is consistent with prior conceptualizations of anxiety, such as those found in gold-standard CBT treatments, but the difference is that we offer a predictive model of how sensory-perceptual and cognitive models of anxiety may arise, well beyond a static belief/filter model often applied in the former (also see Box 1).

**Box 1. Why the free energy principle?**

The free energy principle specifies that the sustainable existence of a biological system is tantamount to it occupying a small number of characteristic (attracting) states. This commitment, we argue, is the free energy principle’s strength compared with Bayesian and reinforcement learning paradigms that do not carry such commitments. In describing the existential parameters and characteristic states to which biological systems are driven, the free energy principle elaborates on those states that an organism would attempt to avoid in acting on their world model. If these states cannot be met (i.e., free energy cannot be reduced) the organism’s internal model of the external must by virtue exude a higher degree of (information theoretic) entropy (see Hirsh et al., 2012).

The application of the principle to general theories of the brain is illustrated by placing (information theoretic) entropy within a probability distribution—in which the “flatness” of the distribution is equivalent to the organism’s model estimates of the uncertainty of its future states, based on past and current sensory data. If the organism has a more precise prediction of its future states, and a high precision belief that these predictions are accurate, then the distribution becomes increasingly pointed. Cognitively, this means the agent has a clear idea as to the causes of sensory data and is confident that acting upon their current beliefs will enable them to minimize uncertainty. On the other hand, if the probability distribution is flat then the agent has a less precise prediction of its future states (see Clark et al., 2018).

By way of example, we can state that sufficiently long-lasting surprise necessarily leads organisms to anticipate a greater degree of surprise. In other words, if we are wrong about what action to take to minimize free energy all of the time, we will form a posterior belief of expected future error (i.e., learned helplessness) whereas if we are right all of the time, we are left with the expectation of no future error; neither of these hypotheticals are tenable in real-world cognition; however. If we are right half of the time and wrong half the time, our model is not left with clear directionality for future prediction, laying the path for potential uncertainty at a meta-prediction level. This necessarily leads to a flat distribution regarding both future predictions and the expected error of those predictions. Given a 50-50 (or overly variable) distribution, we are left with a lack of precision as to what to act on, regardless of prior preferences. Predictions regarding the future can be neither expectant nor non-expectant of error states. Recast as informational (Shannon) surprise, we are left with the *most* anticipated uncertainty for the future. In this way, we can state that even in individual iterations in which one outcome is clearer than others, that if given a particular period of uncertainty, the model can still learn to expect uncertainty in its future, given past volatility.

The free energy principle offers a foundational account of what agents must be driven toward, given the parameters of the physical world which they inhabit. Available free energy thus provides a kind of “rules of the game” the organism must play by to sustain their own existence. We can assume the core motivation to minimize free energy will remain the same irrespective of the anxious pathology. For example, the fact that agents are still guided toward the minimization of free energy may help explain the cyclical feedback response in which anxiety can produce further anxiety, such as the classic case in which entering a situation where panic or anxiety has been learned leads to subsequent anticipation of anxiety upon re-entering that situation, which provokes further anxiety (Clark, 1986). An arguably radical interpretation of our view would suggest that this core motive toward the minimization of free energy manifests as a literal fear of dissipation and destruction, similar to what is specified in psychological theories of mortality aversion such as terror management theory (Greenberg et al., 1986). Note that our account does *not* make this claim. Still, the notion that learned uncertainty and the fear of death may interact in interesting and important ways may be an area worthy of future conceptual clarification.

### With uncertainty equated, why doesn’t anxiety develop in everyone?

Why should anxiety disorder develop in some, but not all, when uncertainty is equated between individuals (Zuckerman, 1999)? We agree that not all individuals will have the same likelihood of developing anxiety disorder, even in the face of an equally uncertain environment. The diathesis stress model provides a popular account to explain such variability, suggesting pathologies arise *via* mutual feedback between genetic predispositions combined with environmental stressors (Zuckerman, 1999). This implies that an individual with any given genetic “set” will interact differently with their environment (Schiele and Domschke, 2018), and thus differing degrees of uncertainty (or stressor events) will be needed for pathological anxiety to form (Frank et al., 2006). Further, protective environmental factors such as a stable and safe family environment, supportive relationships, facets of culture and religion, and the presence of role models can offset or “buffer” against the experienced uncertainty which would otherwise result in subsequent pathology (Tyler et al., 2018). We suggest that these factors will protect against the formation of those internalized metacognitive beliefs regarding the individual’s inability to alter the probability of a future outcome. Because our account specifies that this metacognitive belief formation is intrinsic to the development of pathological anxiety, this provides a tentative rationale for why anxiety disorder will develop in some—but not all—when uncertainty is equated. Despite this, the experience, modeling, and learning of uncertainty itself, remains what underpins the development of anxiety disorder between individuals.

In a way, then, our response to this question is that its premise—namely, that uncertainty can be equated between individuals—is fundamentally flawed: given said background factors, we argue no two situations are ever *truly* equal. If it were possible to measure the internal entropy or free energy of a biological system, then this may actually be testable. It turns out that central nervous system arousal is one of several candidate markers of the physiological equivalent to entropy (Quinkert et al., 2011; Carhart-Harris et al., 2014). Notably, arousal and other candidate markers are readily measurable, for example *via* electroencephalogram and near-infrared spectroscopy (Keshmiri, 2020). Hence, a “measure” of the internal entropy or free energy of relevant biological systems (i.e., clinical patients) may—at least theoretically—be possible, which would provide a means to test between-subject differences in uncertainty tolerance.

## Summary and Conclusion

In this paper, we have endeavored to account for how anxiety forms *via* the free energy principle. From principles derived from the free energy framework, the formation of anxiety and anxiety disorder can be understood as a process of learned uncertainty. Conceptualizing anxiety formation in this fashion situates its genesis at a fundamental principles level and provides a solid grounding to understand the necessary conditions for how and why anxiety develops in the first place. Humans must be psychologically motivated toward characteristic states—those attracting states that sustain existence. When the degree of uncertainty within these states persists for long enough, the organism’s generative world model specifies that this uncertainty is inherent to the perceived world. The agent is left with expecting uncertainty in actively updating its world model, which may cascade to the detriment of the agent’s psychological health. We invite researchers to build on our speculations, with specific reference to questions yet unanswered regarding protective factors, vulnerability factors, and possible treatment and remediation options.

## Data availability statement

The original contributions presented in this study are included in the article/supplementary material, further inquiries can be directed to the corresponding author.

## Author contributions

HM conceived the project with supervision by BH and AD. PC, PL, and KB provided review and intellectual contributions. BH, HM, AD, and HB drafted the manuscript. All authors contributed to the article and approved the submitted version.
